# Enhanced Conversion Efficiencies in Dye-Sensitized Solar Cells Achieved through Self-Assembled Platinum(II) Metallacages

**DOI:** 10.1038/srep29476

**Published:** 2016-07-11

**Authors:** Zuoli He, Zhiqiang Hou, Yonglei Xing, Xiaobin Liu, Xingtian Yin, Meidan Que, Jinyou Shao, Wenxiu Que, Peter J. Stang

**Affiliations:** 1 Electronic Materials Research Laboratory, International Centre for Dielectric Research, Key Laboratory of the Ministry of Education, School of Electronic and Information Engineering, State Key Laboratory for Manufacturing Systems Engineering, Xi’an Jiaotong University, Xi’an 710049, People’s Republic of China; 2 Department of Chemistry, University of Utah, 315 South 1400 East, Room 2020, Salt Lake City, Utah 84112, United States

## Abstract

Two-component self-assembly supramolecular coordination complexes with particular photo-physical property, wherein unique donors are combined with a single metal acceptor, can be utilized for many applications including in photo-devices. In this communication, we described the synthesis and characterization of two-component self-assembly supramolecular coordination complexes (SCCs) bearing triazine and porphyrin faces with promising light-harvesting properties. These complexes were obtained from the self-assembly of a 90° Pt(II) acceptor with 2,4,6-tris(4-pyridyl)-1,3,5-triazine (TPyT) or 5,10,15,20-Tetra(4-pyridyl)-21H,23H-porphine (TPyP). The greatly improved conversion efficiencies of the dye-sensitized TiO_2_ solar cells were 6.79 and 6.08 respectively, while these SCCs were introduced into the TiO_2_ nanoparticle film photoanodes. In addition, the open circuit voltage (Voc) of dye-sensitized solar cells was also increased to 0.769 and 0.768 V, which could be ascribed to the inhibited interfacial charge recombination due to the addition of SCCs.

Due to low cost and highly efficient conversion of photovoltaic energy, numerous research has been studied to improve photovoltaic characteristics as well as inquiry understand the underlying electron-transfer processes in dye-sensitized solar cells (DSSCs) recently^1^,^2^,^3^,^4^,^5^. Usually, the conventional DSSCs consist of the photoanode, the counter electrode, and the liquid electrolyte which often contains i.e. iodide(I^−^)/tri-iodide(I^3−^) redox couples^6^,^7^. The photoanode is usually composed of nanocrystalline semiconductor oxide particle films, and the counter electrode is usually composed of Pt-coated or other noble-metal coated conducting substrates. In DSSCs, the dye molecules will capture photons from the incident light, and subsequently the photo-generated electrons excited from the dye molecules (such as N719, which is an efficient dye in DSSCs as reported) will be rapidly injected into the conduction band of the semiconductor oxide particle films photoanode^6^,^7^. The electrons are then extracted in the electrolyte and migrated to the counter electrode. During this process, the holes will be captured by iodide ions and transferred to the counter electrode in the oxidized state, i.e., tri-iodide ions. Finally, recombination of the electron and hole will occur together with regeneration of tri-iodide to iodide ions at the counter electrode, thus, resulting in photo-generated current in the closed loop. Base above, the conversion efficiency of a DSSC is determined by three factors, including light-harvesting efficiency, electron injection and collection efficiency^8^. In recent years, synthetic approaches such as structural modification, surface treatment of mesoscopic oxide films, and use of sensitizers have been taken to improve the performances of DSSCs^9^,^10^,^11^,^12^. It can be indicated that the possibilities to increase the surface area of the semiconductor oxide photoanode (so that more dye molecular could be adsorbed directly on the surface of semiconductor oxide nanoparticles film and simultaneously be reacted with a redox electrolyte directly and effectively) have been successfully attempted. For example, anatase TiO_2_ NP film photoanode to enhance interconnection by using a post-treatment with TiCl_4_ solution or addition a scattering layer were typically used to increase the roughness of the surface and enhance the charge transformation^12^,^13^,^14^,^15^,^16^,^17^,^18^,^19^.

Several attempts to develop alternative organic–inorganic hybrid materials for DSSCs have been made. Such as, Metal–organic frameworks (MOFs) are organic–inorganic hybrid materials consisting of an infinite exchange, network of metal centers (or inorganic clusters) bridged by organic linkers through metal–ligand coordination bonds. Sometimes, MOFs have excellent applications in adsorbent, ion conduction, separation, bio-activity and photo-related areas, which may give a chance to introduce into DSSCs^20^,^21^,^22^,^23^. Furthermore, for the 3-dimensional network structures of MOFs, their design ability enabling accurate material design based on the diversity of combinations could provide the opportunities to develop new functional structured MOFs. Also, some researchers have introduced unique visible-light-responsive MOFs whose organic linkers act as light-harvesting units into the DSSCs. Kundu *et al*.^24^ reported rod-shaped and hexagonal column shaped ZnO microparticles obtained from one-step thermolysis of porous homochiral MOFs. These ZnO microparticles show permanent porosity and visible light emission centered at 605 or 510 nm, when used as photoanodes, the conversion efficiency of DSSCs were 0.15% and 0.14%, respectively. Bella *et al*.^25^ described the preparation of a polymer composite containing an Mg-MOF through a rapid and environmentally friendly UV-induced free-radical process. Notable solar energy conversion efficiencies were obtained (4.8%), when the composite was used as an electrolyte for DSSCs. Li *et al*.^26^ investigated the influence of coating a TiO_2_ electrode with MOFs on the performance of DSSCs for the first time. In this case, its conversion efficiency was improved by 4.5% (from 5.11 to 5.34%), and the enhancement of the open circuit voltage (Voc) of DSSCs was ascribed to retarded interfacial charge recombination. Furthermore, Li *et al*.^27^ also reported that a MOF was screen printed on a ZnO electrode thus hierarchical ZnO parallelepipeds were obtained after calcination, which acts as an effective light scattering layer in dye-sensitized solar cells, leading to significantly improved solar energy conversion efficiencies (3.67%). Hus *et al*.^28^ also fabricated a highly efficient Pt-free dye-sensitized solar cell (DSSC), which its counter electrode was made of cobalt sulfide (CoS) nanoparticles synthesized via surfactant-assisted preparation of a MOF, the small size nanoparticles they obtained increased roughness factor and surface area, hence, enhancing interaction with dye molecules. The enhanced V_oc_ value of the CoS-based DSSCs had greater fill factor value, thus leading to an improved efficiency of 8.1%.

As one knows, the spontaneous formation of metal–ligand bonds is the basis of a well-established methodology for the construction of supramolecular coordination complexes (SCCs) via coordination-driven self-assembly process. SCCs as another kind of metal–organic complexes encompass discrete systems in which meticulously selected metal centers through self-assembly with ligands containing multiple binding sites oriented with specific angularity to generate a finite supramolecular complex^21^,^29^. SCCs can employ rigid organic ligands as structural linkers, and these organic linkers can be usually modified with functional groups that can be meticulously chosen not to interfere with self-assembly process. Once the structures are formed, these functional groups can provide opportunities to impact unique properties that are relative to the functionalized materials^21^,^29^,^30^,^31^,^32^,^33^,^34^,^35^. That is to say, SCCs with promising light-harvesting or electronic properties will have possibilities to improve the performance of DSSCs^36^.

Herein, we created a simple model for the application of SCCs in the field of DSSCs. For the first time, two SCCs with unique optical properties, which were obtained from the self-assembly of a 90° Pt(II) acceptor with 2,4,6-tris(4-pyridyl)-1,3,5-triazine (TPyT) or 5,10,15,20-Tetra(4-pyridyl)-21H,23H-porphine (TPyP) as reported in our previous research^37^,^38^, was dropped on an TiO_2_ nanoparticle (NP) film during fabrication of DSSCs as shown in Fig. 1a. Therefore, enhanced light-harvesting and unique optical properties of these SCCs from their triazine and porphyrin faces were introduced into the TiO_2_ nanoparticle (NP) film photoanodes, thus, resulting in the greatly improved performance in dye-sensitized TiO_2_ solar cells.

The SCCs with particular photo-physical properties can be utilized for the DSSCs applications in photo-devices. First, we described the synthesis and characterization of cage-like SCCs with promising light-harvesting and unique optical properties. These cages were obtained from the self-assembly of a 90° Pt(II) acceptor with 2,4,6-tris(4-pyridyl)-1,3,5-triazine (TPyT) or 5,10,15,20-Tetra(4-pyridyl)-21H,23H-porphine (TPyP) as shown in Fig. 2. All SCCs were characterized by ^1^H and ^31^P multinuclear NMR spectroscopy and ESI mass spectrometry (ESI-MS), confirming the structure of each self-assembly and the stoichiometry of the formation. The two-component SCCs 4 were obtained from the self-assembly of the cis-90° Pt(II) acceptor 3 with TPyT donor 1 in a 3:2 ratio in acetone. In the ^31^P{^1^H} NMR spectrum of the self-assembly SCCs 4 solution, there is only one intense single peak located at 0.33 ppm with concomitant ^195^Pt_satellites_ as shown in Fig. 3a. Likewise, the ^1^H NMR spectrum (Figure S3 in supporting information) shows sharp signals assigned to the coordinated pyridyl moieties from triazine (δ = 9.64 ppm, H_α-Py_; δ = 8.94 ppm, H_β-Py_). These ^1^H NMR spectral results are in accord with the highly symmetric structures of octahedron SCCs 4 bearing triazine faces. ESI mass spectrometry was used here to further confirm the [6+4] self-assembly of 4: concomitant signals are located at m/z = 1725.9 ([4–3OTf]^3+^), 1257.1 ([4–4OTf]^4+^), and 975.9 ([4–5OTf]^5+^). By the way, these signal peaks agree well with their theoretical distributions (shown in Figure S4 in supporting information). NMR results indicate that an acetone solution of the self-assembly SCCs 4 has a diffusion coefficient of (5.37 ± 0.13) × 10^−6^ cm^2^/s at room temperature. Both NMR and ESI-MS support the formation of 4 as the dominant product with a yield of 95% in solution is obtained by precipitation using ethyl ether.

The two-component SCCs 5 were also prepared from the self-assembly of the cis-90^o^ Pt(II) acceptor 3 with TPyP donor 2 in a 6:3 ratio in CD_2_Cl_2_/CD_3_NO_2_ solution. The CD_2_Cl_2_ suspension of tetratopic pyridyl ligand 2 was added a CD_3_NO_2_ solution of the cis-Pt(PEt_3_)_2_(OTf)_2_ acceptor 3 by dropwise with strong stirring. The reaction solution was stirred at room temperature for 1 h and then increased to 70 °C keeping for overnight. Followed that the solution was evaporated to dryness and the SCCs 5 was collected by precipitation using ethyl ether, in the Yield of 95%. The as-obtained SCCs 5 were also characterized by ^31^P and ^1^H multinuclear NMR spectroscopy and ESI mass spectrometry measurements to confirm the formation. In the ^31^P NMR spectra (Fig. 3b), only one intense singlet (0.90 ppm) with concomitant ^195^Pt_satellites_ could be found. And, the ^1^H NMR spectra (See Figure S5 in supporting information) show sharp signals assigned to the coordinated pyridyl moieties from porphine (e.g., δ = 9.75 H_α-Py_, δ = 8.98 H_β-Py_ and H_Pyrrole_, δ = 8.51 H_Pyrrole_, δ = 2.32 PCH_2_CH_3_). These ^1^H NMR spectral results are in accord with the highly symmetric structures of trigonal prism SCCs 5 bearing porphyrin faces. ESI mass spectrometry further confirms the [6+3] self-assembly of 5 (see Figure S6 in supporting information): Signals at m/z = 2966.57 [5–2OTf]^2+^, m/z = 1408.99 [5–4OTf]^4+^, and m/z) 1097.48 [5–5OTf]^5+^. All of these peaks are isotopically resolved and agree well with their theoretical distributions. By the way, a molecular dynamics simulation using Maestro and Macromodel with a MMFF or MM2^*^ force field at 300 K in the gas phase was applied to equilibrate each SCCs, and the output of the simulation was then minimized to full convergence. As shown in Fig. 4, models of SCCs 4 and 5 have the shapes of octahedron and tetragonal prisms, respectively.

The UV–vis spectra of TPyT, TPyP, cis-Pt(PEt_3_)_2_(OTf)_2_ and SCCs were also measured using a Hitachi U-4100 Spectrophotometer. As shown in Fig. 5, The primary characteristic bands of cis-Pt(PEt_3_)_2_(OTf)_2_ were located at 240, 262 and 320 nm; the primary characteristic bands of TPyT and TPyP was located at 248 and 415 nm, respectively. After *coordination-driven self-assembly* with cis-Pt(PEt_3_)_2_(OTf)_2_, the primary characteristic bands of TPyT show a red shift from 248 to 350 nm in its corresponding SCCs 4, and that of show a little red shift from 415 to 425 nm in its corresponding SCCs 5. But it also should be noted, in these two-component SCCs I, the absorbance of main primary characteristic band of cis-Pt(PEt_3_)_2_(OTf)_2_ at 240 nm decreased as shown in Fig. 5.

The SCCs 4 and 5 solutions were dropped on a TiO_2_ nanoparticle (NP) films (The XRD pattern and SEM images of TiO_2_ nanoparticle NP films was present in Figures S7 and S8) photoanodes after being immersed in a 5 × 10^−4^ M N719 dye solution in a mixture of acetonitrile and tert-butanol (1:1, volume ratio of acetonitrile and tert-butanol) for 24 h, and the as-obtained photoanodes were then assembled with Pt/FTO used as counter electrodes by using heat-sealing film, and a liquid electrolyte was injected into interelectrode gap from the holes in counter electrode as shown in Fig. 1. The current-voltage (J-V) characteristics of the ligands or SCCs and dye co-sensitized DSSCs were measured under an illumination of AM 1.5 solar simulator of 100 mW·cm^−2^. The current-voltage (J-V) curves for the as-fabricated DSSCs are shown in Fig. 6, also the details of J-V parameters extracted from the J-V curves, such as the open circuit voltage (V_oc_), I_sc_, J_sc_, I_max_, V_max_, P_max_, FF and the conversion efficiency (η, calculated according to J_sc_ × V_oc_ × FF/(100 mW·cm^−2^) are presented in Table 1. It indicates that the conversion efficiency of N719-sensitized TiO_2_ solar cells without ligands or SCCs is 4.83%. While co-sensitized with the TPyT and TpyP, the conversion efficiencies of the DSSCs increase to 6.07% and 5.05%, respectively. This enhancement is attributed to the increased J_sc_ resulting from the enhanced light-harvesting properties of TPyT and TPyP. During the measurement, the open circuit voltage (V_oc_) will arrive the max after 20 min (Figures S9 and S10 and Table S1 in supporting information, Figure S9 and Table S1 present the details photovoltaic performances of the TPyT/N719 co-sensitized solar cells), which main because inhibited interfacial charge recombination and enhanced electrons transport, which caused by TPyT and TPyP react with I^−^. However, when added the cis-90° Pt(PEt_3_)_2_(OTf)_2_ will react with tert-butylpyridine and break the N719 dye molecular, thus result in the conversion efficiency decreases to 2.93% together with a decrease of J_sc_ from 10.5 to 5.2 mA/cm^2^. The greatly improved conversion efficiencies of the dye-sensitized TiO_2_ solar cells are 6.79% and 6.08%, respectively, while the SCCs 4 and 5 are introduced as co-sensitizer with N719 sensitized TiO_2_ nanoparticle film photoanodes. Some enhancements could be attributed to enhanced light- harvesting and unique optical properties of these SCCs from their triazine and porphyrin faces as mentioned before. Also the enhanced electrons transport behavior caused by TPyT and TPyP reacted with I^−^, will take an important part. In addition, the open circuit voltage (V_oc_) of the dye-sensitized solar cells is also increased to 0.769 and 0.768 V, which is related to the inhibited interfacial charge recombination due to the introduction of the SCCs^9^. By the way, it should be mentioned here that the SCCs 4 and SCCs 5 co-sensitized with N719 show a slow reduction rate due to the *coordination-driven self-assembly process* (Figure S11 in supporting information). When irradiated with the solar light, the temperature will be increased and thus the coordination bond will be broken; the SCCs will be formed again by the coordination-driven self-assembly process when cooling down to room temperature.

Incident photon-to-current conversion efficiency (IPCE) measurements were conducted to analyze the details of the enhanced performance of these ligands or SCCs and dye co-sensitized DSSCs. The IPCE, which corresponds to the external quantum efficiency, is given by


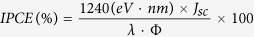


where J_sc_ (mA·cm^−2^) is the short-circuit photocurrent density obtained under monochromatic irradiation and λ(nm) and Φ (mW·cm^−2^) are the wavelength and intensity of the monochromatic light, respectively^39^. Figure 7 shows action spectra of IPCE for DSSCs based on the TiO_2_ NP film electrodes (ca. about 10 μm thickness) sensitized with N719, TPyT/N719, TPyP/N719, Pt(II)/N719, SCCs 4/N719 and SCCs 5/N719, respectively. The IPCE is determined by quantum yield of the electron injection, light absorption efficiency of the dye molecules, and efficiency of collecting the injected electrons at the conducting glass substrate, which is strongly affected by the structure and phys-chemical property of the photoelectrodes^14^,^40^. The improved J_sc_ of the TPyT/N719 and TPyP/N719 co-sensitized solar cell is due to the enhanced light-harvesting properties of the TPyT and TPyP, which can be seen in the related IPCE spectra between 400–700 nm. Contrary to this, an improved IPCE value of the cis-90° Pt(II)/N719, SCCs 4/N719 and SCCs 5/N719 co-sensitized solar cell at short wavelength region as shown in Fig. 7b is attributed to the cis-90° Pt(II) accepter, which also indicates that some of SCCs will break down and the cis-90° Pt(II) accepter will exist in the DSSCs after compared with their absorption spectra. Among these DSSCs, the SCCs 4/N719 and SCCs 5/N719 co-sensitized solar cells show higher IPCE value (reached 55% at 526 nm and 53% at 515 nm, respectively) than that of the TPyT/N719 and TPyP/N719 co-sensitized ones, which results from the enhanced light-harvesting properties of these SCCs from their triazine and porphyrin faces^29^,^36^,^41^,^42^. Furthermore, some more reactions mechanism will be needed to research in future.

In summary, we have successfully synthesized two-component self-assembly supramolecular coordination complexes (SCCs) bearing triazine and porphyrin faces with promising light-harvesting properties. When the as-synthesized SCCs were dropped on the TiO_2_ nanoparticle film photoanodes, there is a greatly improved conversion efficiency of the as-obtained dye-sensitized TiO_2_ solar cells, which can be attributed to the enhanced light-harvesting and unique optical properties of the SCCs from their triazine and porphyrin faces. It is also noted that the open circuit voltages (Voc) of the dye-sensitized solar cells increase to 0.769 and 0.768 V, which should be related to the inhibited interfacial charge recombination due to the addition of the SCCs. The preliminary results indicate that the SCCs are promising materials for improving the conversion efficiency of DSSCs. We believe that the presented results here will provide a novel approach for improving the photovoltaic properties of DSSCs. Meanwhile, present study may open up a new application field for SCCs.

## Experimental Section

### Materials

cis-Pt(PEt_3_)_2_(OTf)_2_ (3) was prepared according to literature procedures^43^. All other compounds such as 5,10,15,20-Tetra(4-pyridyl)-21H,23H-porphine (TPyP) were bought from Sigma-Aldrich, whereas the solvents were purchased from Cambridge Isotope Laboratory.

### Synthesis of the trigonal 3-connector 2,4,6-tris(4-pyridyl)-1,3,5-triazine(TPyT)

18-crown-6 (500 g, 1.9 mmol) and KOH (112.5 mg, 2.0 mmol) were dissolved in 10 mL ethanol and stirred for 20 min. The solution was concentrated to remove solvent under reduced pressure and got an oil product. To this oil, 5 g of 4-cyanopyridine was added. The mixture was heated at 200 °C. The color changed to red within a short period of time. After stirring at this temperature for 6 h, the mixture was cooled to room temperature, and then 60 mL pyridine was added. Stirring the mixture for 5 min gave the fine crystal compound that was filtrated off and washed with pyridine (25 mL twice) and toluene (25 mL). The pale-red crystals were dissolved in 2 mol L^−1^ HCl (60 mL). After removing small amount solid by filtration, ad*J*usting the solution to slight alkaline using concentrated NH_3_ aqueous gave a pale-white powder that was filtrated, washed with water, and dried in air to afford 3.5 g TPT (yield: 70%), ^1^H NMR(CD_2_Cl_2_): δ 8.92 (6H), δ 8.60 (6H).

### Self-Assembly of 4

SCCs 4 was prepared according to literature procedures^37^. Cis-Pt(PEt_3_)_2_(OTf)_2_ (3) (6.08 mg, 8.34 μmol) and tripyridyl ligand 1 (1.67 mg, 5.35 μmol) were placed in a 2-gram vial, followed by addition of 0.8 mL of acetone-d6, and the vial was then sealed with Teflon tape and immersed in an oil bath at 70 °C for 3 h. The [6+4] self-assembly of starting metal-organic supramolecule 4 was obtained, and the solid product was isolated by addition of ethyl ether. Yield: 95% (7.2 mg). ^1^H NMR (acetone-d6, 300 MHz) δ 9.64 (d, J_1_ = 5.1 Hz, 24H, H_α-Py_), 8.94 (d, J_1_ = 6.3 Hz, 24H, H_β-Py_), 1.96 (m, 72H, PCH_2_CH_3_), 1.23 (m, 108H, PCH_2_CH_3_). ^31^P{^1^H} NMR (acetone-*d6*, 121.4 MHz) δ 0.33 (s, ^195^Pt _satellites_, ^1^J_Pt-P_ = 3085 Hz). MS (ESI) calcd for [M-3OTf]^3+^ m/z = 1726.0, found 1725.9; calcd for [M-4OTf]^4+^ m/z = 976.0, found 975.9. Anal. Calcd for C_156_H_228_F_36_N_24_O_36_P_12_Pt_6_S_12_(C_3_H_6_O)_3_: C, 34.16; H, 4.27; N, 5.80. Found: C, 34.44; H, 4.56; N, 5.59.

### Self-Assembly of 5

SCCs 4 was prepared according to literature procedures^38^. 1.2 mL CD_2_Cl_2_ suspension of tetratopic pyridyl ligand 2 (2.49 mg, 4.02 μmol) was added a 0.4 mL CD_3_NO_2_ solution of cis-Pt(PEt_3_)_2_(OTf)_2_ (3) (5.89 mg, 8.07 μmol) dropwise with continuous stirring (6 min). The reaction mixture was stirred at room temperature for 1 h and then heated to 70 °C overnight. The solution was evaporated to dryness, and the product was collected. Yield: 95% (7.96 mg). ^1^H NMR (CD_2_Cl_2_/CD_3_NO_2_ = 3/1, 300 MHz) δ 9.75 (dd, J1 = 4.8 Hz and J2 = 36 Hz, 24H, H_α-Py_), 8.98 (m, 36H, H_β-Py_ and H_Pyrrole_), 8.51 (s, 12H, H_Pyrrole_), 2.32 (m, 72H, PCH_2_CH_3_), 1.60 (m, 108H, PCH2CH3). ^31^P{^1^H} NMR (CD_2_Cl_2_/CD_3_NO_2_ = 3/1, 121.4 MHz) δ 0.90 (s, ^195^Pt _satellites_, ^1^J_Pt-P_ = 3070 Hz). MS (ESI) calcd for [M - 2OTf]^2+^ m/z 2966.54, found 2966.57; calcd for [M-4OTf]^4+^ m/z 1409.04, found 1408.99; calcd for [M-5OTf]^5+^ m/z 1097.44, found 1097.48. Anal. Calcd for C_204_H_258_F_36_N_24_O_36_P_12_Pt_6_S_12_: C, 39.31; H, 4.17; N, 5.39. Found: C, 39.83; H, 4.35; N, 5.16.

### Preparation of TiO_2_ nanoparticle (NP) film photoanode

Viscous TiO_2_ paste, which was prepared by mixing P25, ethanol, terpinol, and ethyl cellulose, was coated on FTO glass by a screen-printing technique. Then, the as-obtained TiO_2_ nanoparticle (NP) film photoanode was immersed into 40 mM TiCl_4_ aqueous solution at 70 °C for 30 min, which is expected to improve the photovoltaic performances of the devices. After this treatment, the photoanode was sintered at 500 °C for 30 min. Finally, the as-obtained photoanode was immersed into a 5 × 10^−4^ M solution of the N719 dye in in a mixture of acetonitrile and tert-butanol (1:1, volume ratio of acetonitrile and tert-butanol) for 24 h, and washed with ethanol and dried for next step.

### Preparation of Pt/FTO glass counter electrode

The standard Pt/FTO counter electrode was prepared by DC sputtering at 6 mA for 10 min, and a Pt thin film of about 20 nm thicknesses on a fluorine-doped SnO_2_ glass substrate was prepared.

### Assembly of the dye-sensitized solar cells

The DSSC device was composed of the N719 sensitized TiO_2_ photoanode coupled with the Pt/FTO glass counter electrode. In this step, 2 drops of ligands or SCCs (5 mM in Acetone) were used to cover surface of N719 sensitized TiO_2_ photoanode. The distance between these two electrodes was fixed and sealed by heating heat-sealing film with a thick of 40 μm. Then the electrolyte, which contains 0.03 M I_2_, 0.6 M 1-methyl-3- propylimidazolium iodide (PMII), 0.10 M guanidinium thiocyanate, and 0.5 M tert-butylpyridine in solutions (15:85 volume ratio of valeronitrile and acetonitrile), was injected into the gap between these two electrodes from one small hole in counter electrode by syring.

### Photovoltaic parameters measurements

The power conversion efficiencies of the as-fabricated DSSCs were obtained under 100 mW/cm^2^ light illumination by a Sol3A all-optical spectrum solar simulator (94043A-type) and the J-V data was collected by the PVIV-1A IV measurement. The incident photon-to-current conversion efficiency of these DSSCs were obtained by QTEST STATION 1000ADX solar cell IPCE test system.

## Additional Information

**How to cite this article**: He, Z. *et al*. Enhanced Conversion Efficiencies in Dye-Sensitized Solar Cells Achieved through Self-Assembled Platinum(II) Metallacages. *Sci. Rep*. **6**, 29476; doi: 10.1038/srep29476 (2016).

## Supplementary Material

Supplementary Information

## Figures and Tables

**Figure 1:**
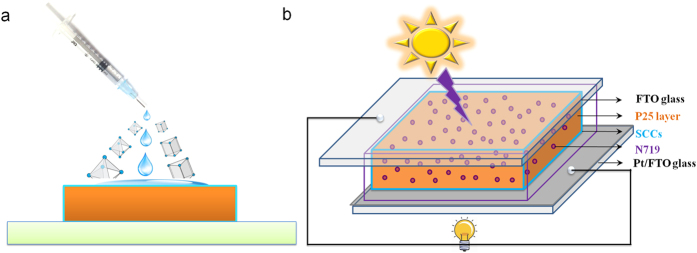
(**a**) Schematic representation of TiO_2_ NP film photoanodes with the addition of SCCs (the blue drop is SCCs solution); (**b**) Design scheme of such SCCs and N719 co-sensitized solar cells.

**Figure 2:**
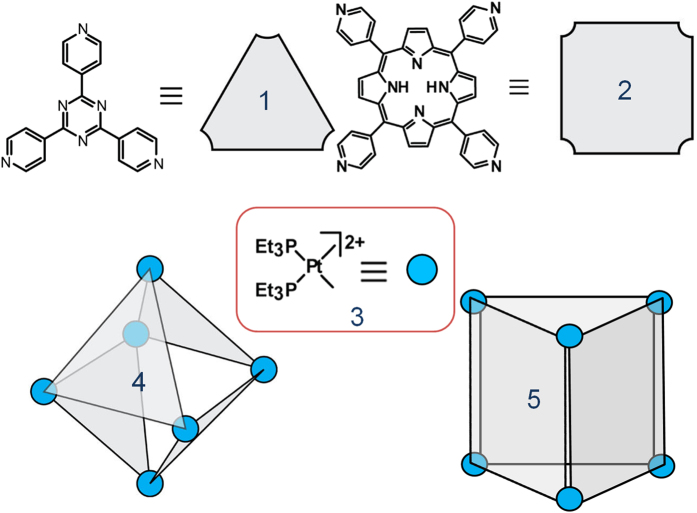
Schematic illustration of self-assembly of supra-molecular coordination complexes 4 and 5.

**Figure 3:**
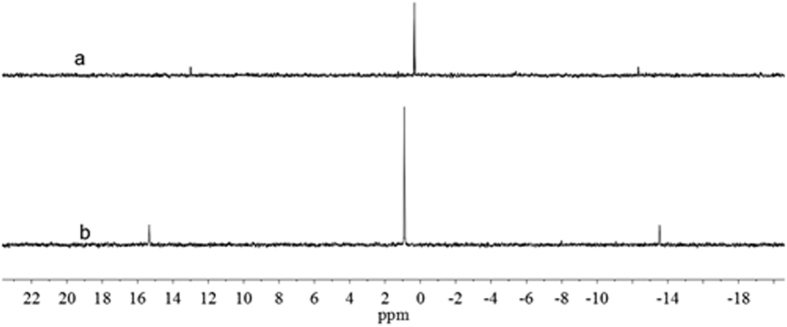
^31^P NMR spectra of the discrete [6+4] SCCs 4 and [6+3] SCCs 5.

**Figure 4:**
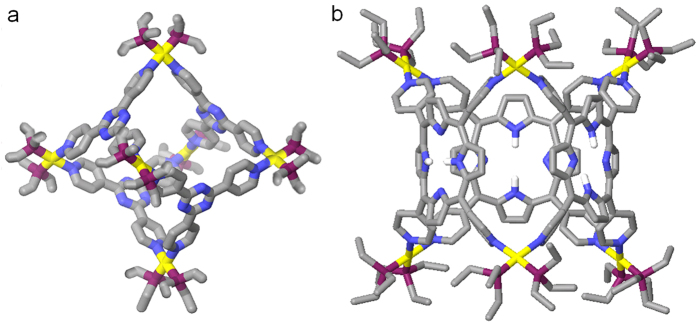
Computational models (MMFF) of (**a**) the discrete [6+4] SCCs 4 and (**b**) [6+3] SCCs 5.

**Figure 5:**
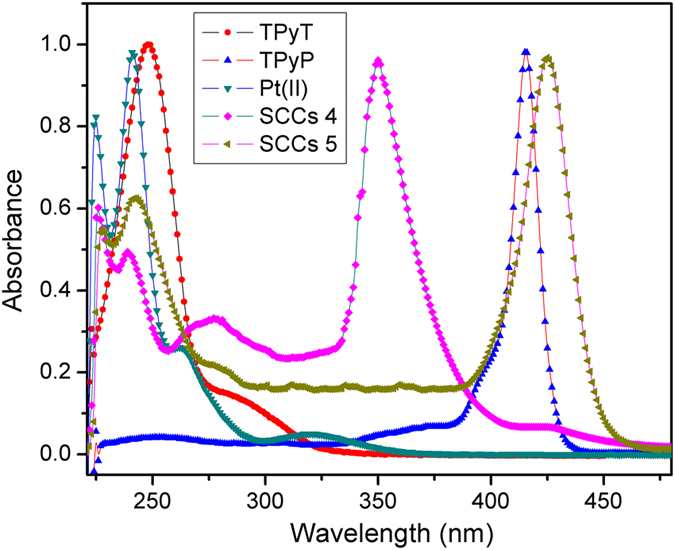
Absorption spectra of the as-obtained ligands and SCCs (5 × 10^−5^ M in DCM).

**Figure 6:**
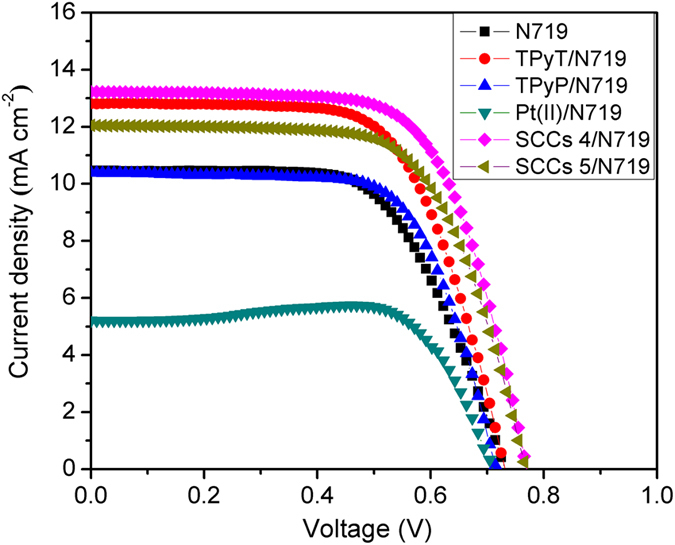
J-V characteristics of the solar cells recorded under AM 1.5G illumination (100 mW/cm^2^) by using ligands or SCCs and dye co-sensitized TiO_2_ NP films as photoanodes.

**Figure 7:**
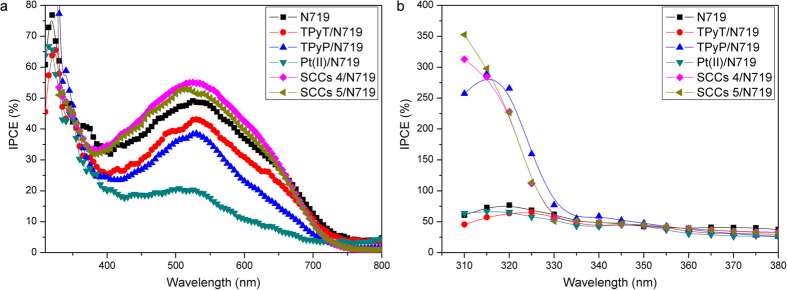
IPCE spectra recorded by using ligands or SCCs and dye co-sensitized TiO_2_ photoanodes.

**Table 1:** Photovoltaic Performances f Solar Cell with ligands or SCCs and Dye as Photosensitizers.

Samples	V_oc_/V	I_sc_/A	J_sc_/mA·cm^−2^	I_max_/A	V_max_/V	P_max_/mW	Fill Factor	Efficiency
N719	0.732	0.00262	10.5	0.00238	0.507	1.206	63.0	4.83
TPyT/N719	0.733	0.00320	12.8	0.00288	0.527	1.518	64.6	6.09
TPyP/N719	0.716	0.00260	10.4	0.00236	0.534	1.264	67.8	5.05
Pt(II)/N719	0.710	0.00130	5.2	0.00136	0.538	0.733	79.5	2.93
SCCs 4/N719	0.769	0.00330	13.2	0.00297	0.572	1.699	73.5	6.79
SCCs 5/N719	0.768	0.00301	12.0	0.00270	0.563	1.520	65.7	6.08
