# Supplementary material for: Enhanced Conversion Efficiencies in Dye-Sensitized Solar Cells Achieved through Self-Assembled Platinum(II) Metallacages
Source: Sci Rep. 2016 Jul 11;6:29476. doi: 10.1038/srep29476 (PMC4941399; doi:10.1038/srep29476)
Supplement: Supplementary Information [file srep29476-s1.pdf]

Enhanced Conversion Efficiencies in Dye-Sensitized Solar Cells Achieved through Self-Assembled Platinum(II) Metallacages

Zuoli He,[a, b] Zhiqiang Hou,[a] Yonglei Xing,[a] Xiaobin Liu,[a] Xingtian Yin,[a] Meidan Que,[a] Jinyou Shao,[a] Wenxiu Que [a, *] and Peter J. Stang [b,*]

a Electronic Materials Research Laboratory, International Centre for Dielectric Research, Key Laboratory of the Ministry of Education, School of Electronic and Information Engineering, State Key Laboratory for Manufacturing Systems Engineering, Xi'an Jiaotong University, Xi'an 710049, People's Republic of China

b Department of Chemistry, University of Utah, 315 South 1400 East, Room 2020, Salt Lake City, Utah 84112, United States

[*] Email address: wxque@xjtu.edu.cn; Stang@chem.utah.edu

Supporting Information (12 Pages)

Figure S1. Molecular structure of N719 dye. 3

Figure S2. ^{1}H NMR spectra of TPT. 4

Figure S3. (a) 31P{1H } and (b) Partial 1H NMR (Acetone-*d6*, 300MHz) spectra of the [6+4] SCCs 4. 5

Figure S4. Full ESI MS spectra of the [6+4] SCCs 4. 6

Figure S5. Partial 1H NMR (CD_3NO_2/CD_2Cl_2 = 1:3, 300MHz) spectrum of [6+3] SCCs 5. 7

Figure S6. Calculated (blue, top) and Experimental (red, bottom) ESI mass spectra of [6+3] SCCs 5. 7

Figure S7 XRD pattern of TiO_2 nanoparticle (NP) film photoanode 8

Figure S8 SEM images of TiO_2 nanoparticle (NP) film photoanode 9

Figure S9 Open circuit voltage (V_{oc}) of the solar cells recorded under AM 1.5G illumination (100 mW/cm^2) by using ligands or SCCs and dye co-sensitized TiO_2 NP films as photoanodes 10

Figure S10 J−V characteristics of solar cell recorded under AM 1.5G illumination (100 mW/cm^2) by TypT/N719 co-sensitized TiO_2 as photoanode. 11

Figure S11 Stability of the ligands or SCCs and dye co-sensitized solar cells recorded under AM 1.5G illumination (100 mW/cm^2) (keep the DSSCs in dark at room temperature) 12

Table S1 Photovoltaic performances of TypT/N719 co-sensitized solar cells. 13

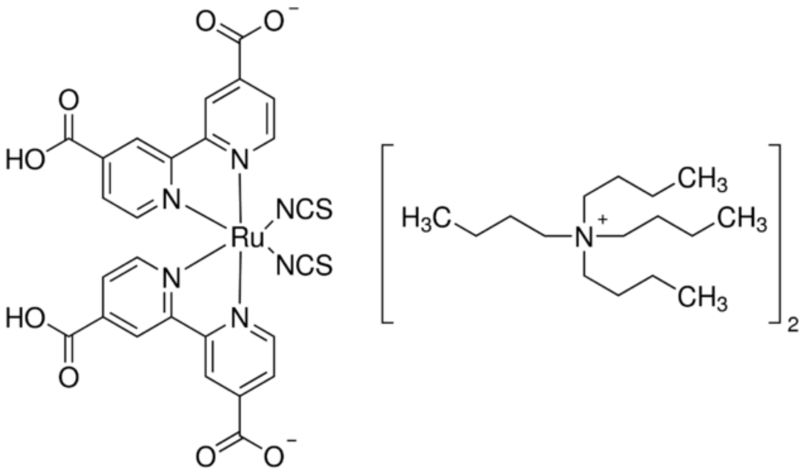

Figure S1. Molecular structure of N719 dye.

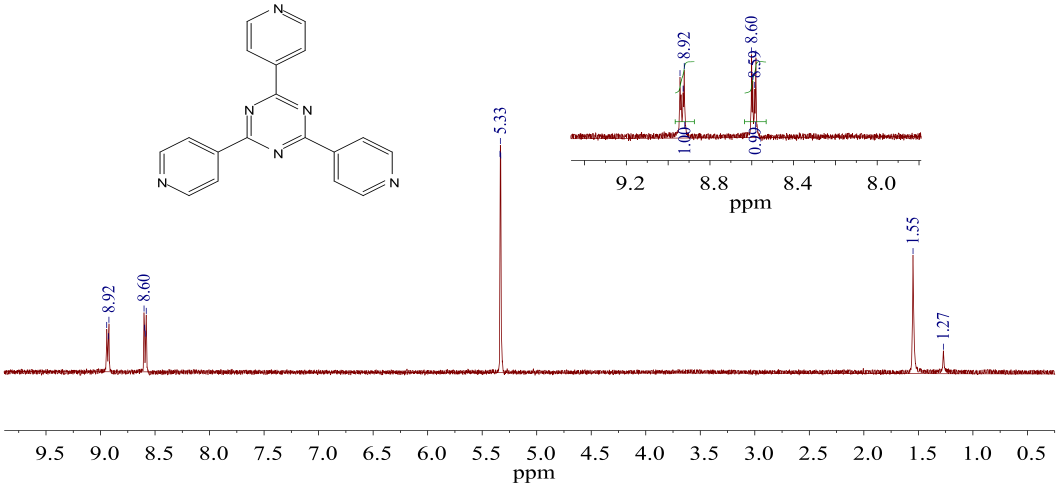

Figure S2. ^{1}H NMR spectra of TPT.

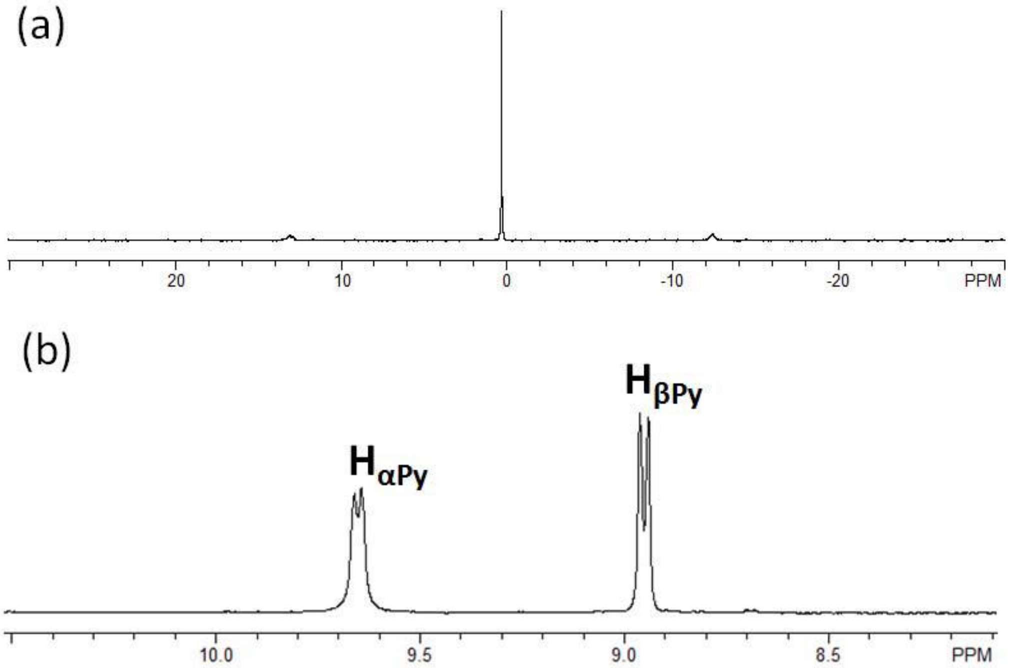

Figure S3. (a) 31P{1H } and (b) Partial 1H NMR (Acetone-*d6*, 300MHz) spectra of the [6+4] SCCs 4.

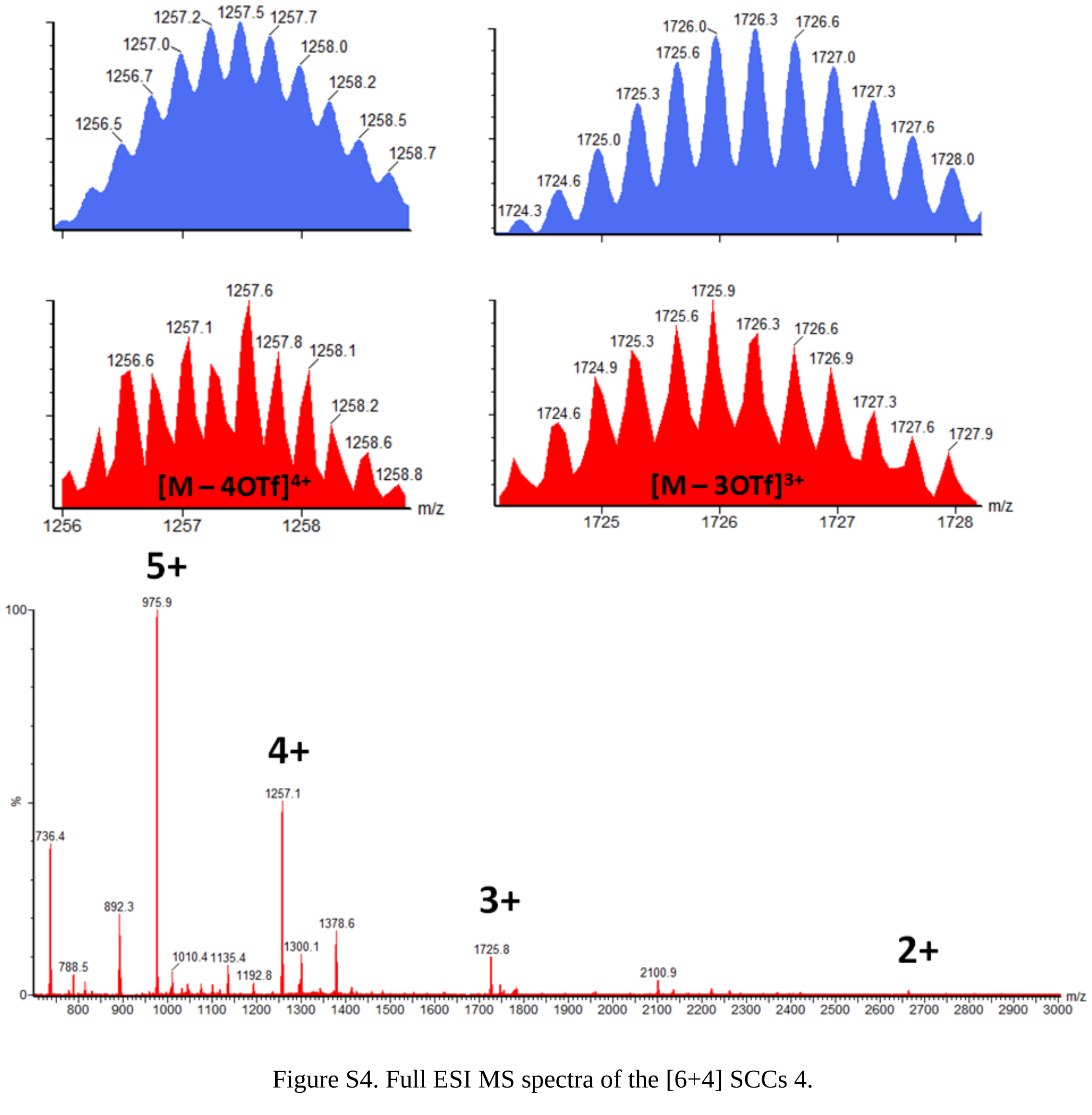

Figure S4. Full ESI MS spectra of the [6+4] SCCs 4.

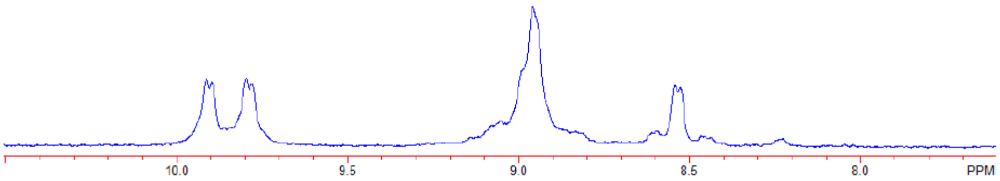

Figure S5. Partial 1H NMR (CD_3NO_2/CD_2Cl_2 = 1:3, 300MHz) spectrum of [6+3] SCCs 5.

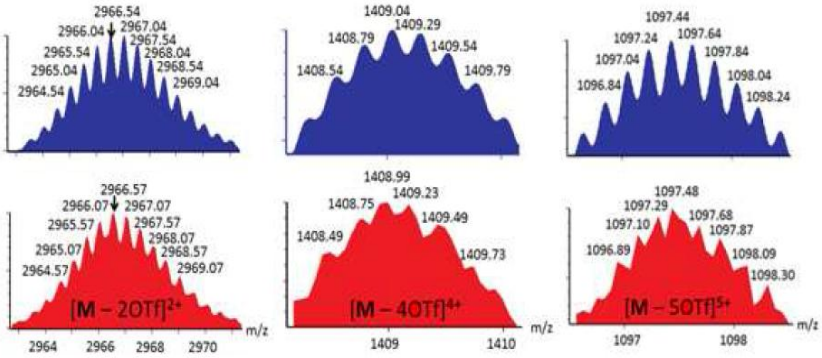

Figure S6. Calculated (blue, top) and Experimental (red, bottom) ESI mass spectra of [6+3] SCCs 5.

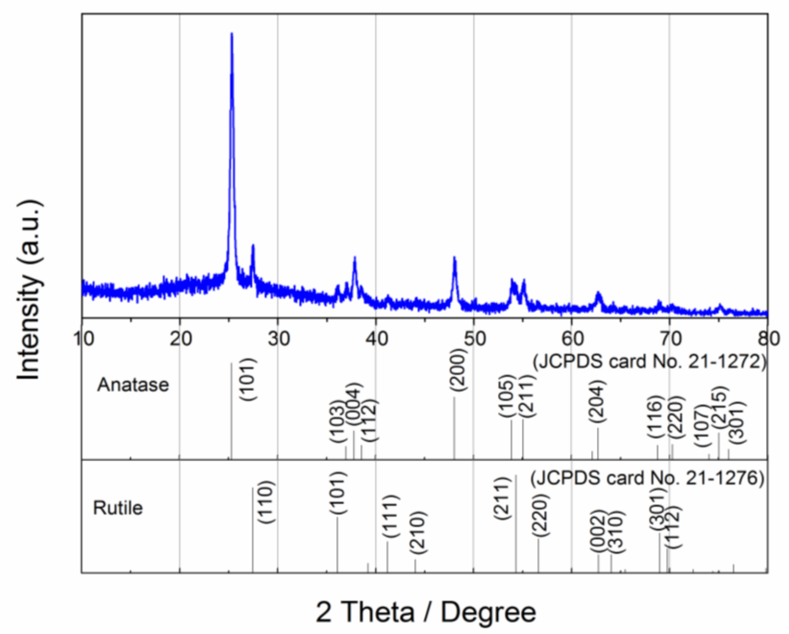

Figure S7 XRD pattern of TiO_2 nanoparticle (NP) film photoanode

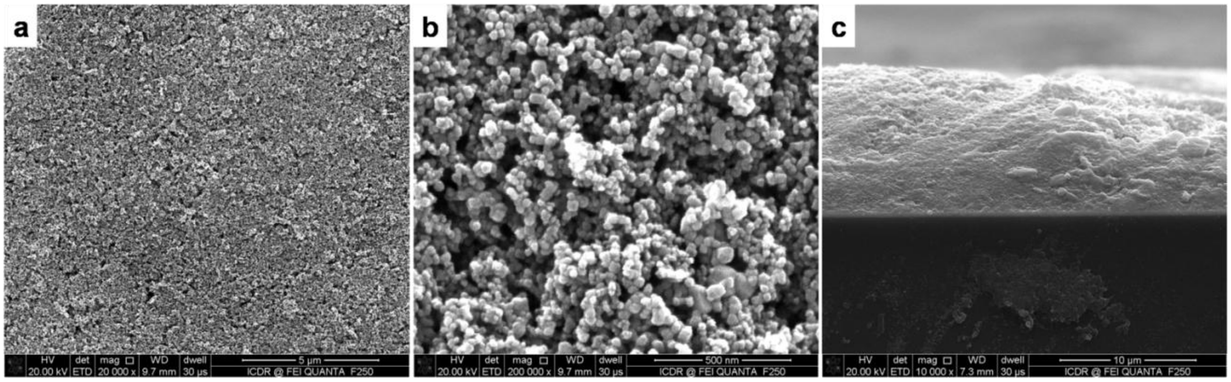

Figure S8 SEM images of TiO_2 nanoparticle (NP) film photoanode

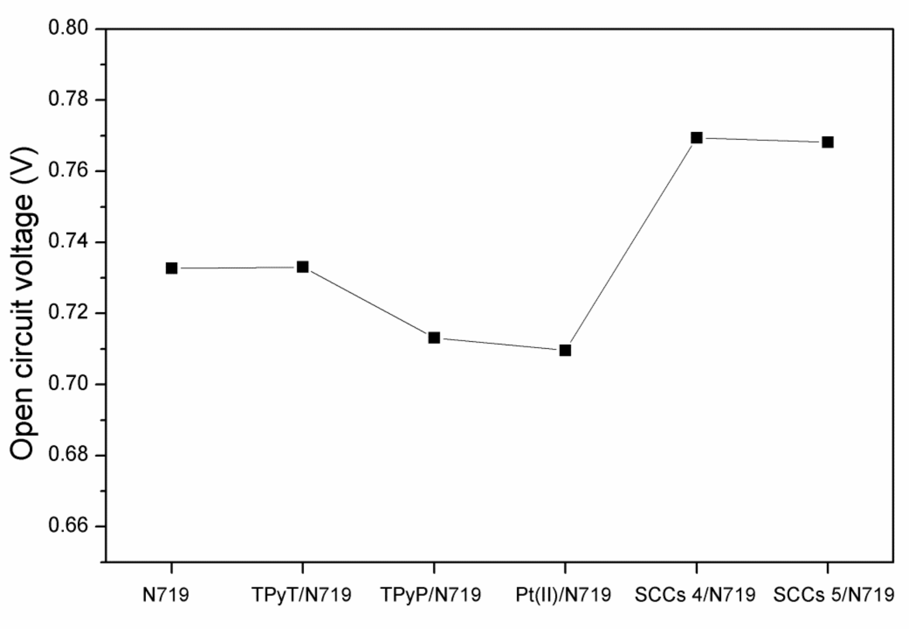

Figure S9 Open circuit voltage (V_{oc}) of the solar cells recorded under AM 1.5G illumination (100 mW/cm^2) by using ligands or SCCs and dye co-sensitized TiO_2 NP films as photoanodes

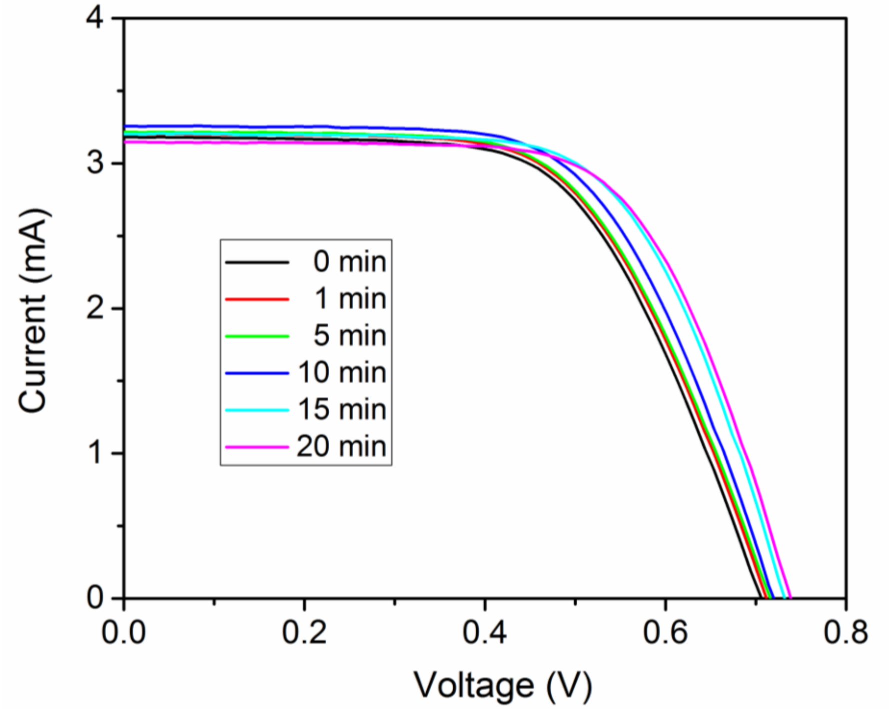

Figure S10 J−V characteristics of solar cell recorded under AM 1.5G illumination (100 mW/cm^2) by TypT/N719 co-sensitized TiO_2 as photoanode.

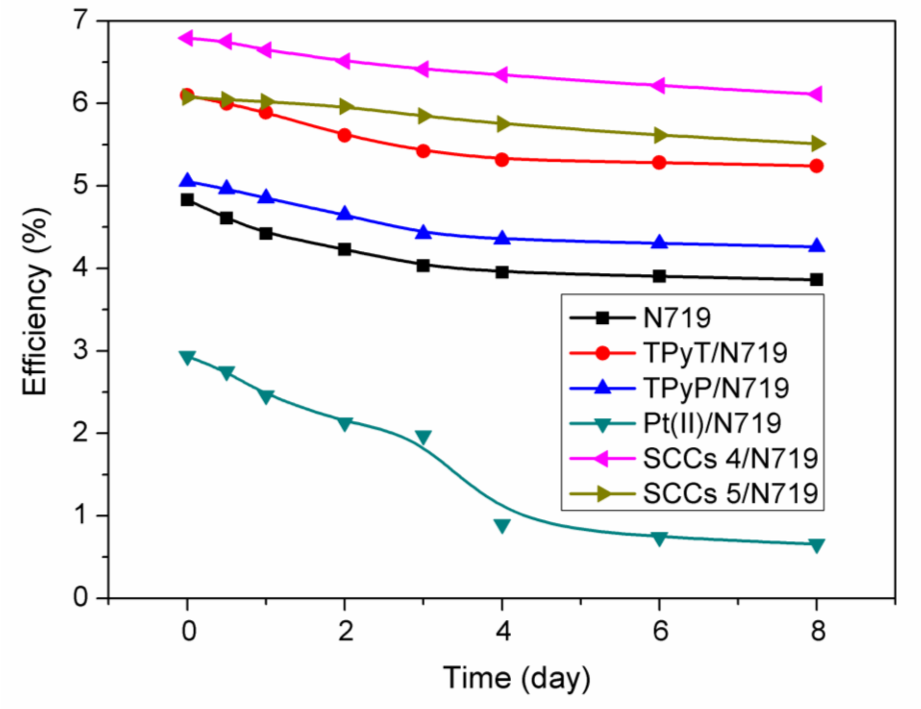

Figure S11 Stability of the ligands or SCCs and dye co-sensitized solar cells recorded under AM 1.5G illumination (100 mW/cm^2) (keep the DSSCs in dark at room temperature)

Table S1 Photovoltaic performances of TypT/N719 co-sensitized solar cells.

	Voc V	Isc A	Jsc mA/cm^2	Fill Factor	Efficiency
0 min	0.70557	0.003182	12.72931	61.2999	5.5056
1 min	0.712491	0.003205	12.81899	61.2682	5.5959
5 min	0.715868	0.003217	12.86978	61.1124	5.6303
10 min	0.720263	0.003258	13.03116	62.2268	5.8405
15 min	0.732864	0.003205	12.81966	64.6457	6.0735
20 min	0.739152	0.003147	12.58715	65.5353	6.0973
